# Isolation of a Novel Phage with Activity against *Streptococcus mutans* Biofilms

**DOI:** 10.1371/journal.pone.0138651

**Published:** 2015-09-23

**Authors:** Marion Dalmasso, Eric de Haas, Horst Neve, Ronan Strain, Fabien J. Cousin, Stephen R. Stockdale, R. Paul Ross, Colin Hill

**Affiliations:** 1 School of Microbiology, University College Cork, Cork, Ireland; 2 APC Microbiome Institute, University College Cork, Cork, Ireland; 3 Department of Microbiology and Biotechnology, Max Rubner-Institut, Kiel, Germany; 4 Teagasc Biotechnology Centre, Moorepark Food Research Centre, Fermoy, Co. Cork, Ireland; University of Kansas Medical Center, UNITED STATES

## Abstract

*Streptococcus mutans* is one of the principal agents of caries formation mainly, because of its ability to form biofilms at the tooth surface. Bacteriophages (phages) are promising antimicrobial agents that could be used to prevent or treat caries formation by *S*. *mutans*. The aim of this study was to isolate new *S*. *mutans* phages and to characterize their antimicrobial properties. A new phage, ɸAPCM01, was isolated from a human saliva sample. Its genome was closely related to the only two other available *S*. *mutans* phage genomes, M102 and M102AD. ɸAPCM01 inhibited the growth of *S*. *mutans* strain DPC6143 within hours in broth and in artificial saliva at multiplicity of infections as low as 2.5x10^-5^. In the presence of phage ɸAPCM01 the metabolic activity of a *S*. *mutans* biofilm was reduced after 24 h of contact and did not increased again after 48 h, and the live cells in the biofilm decreased by at least 5 log cfu/ml. Despite its narrow host range, this newly isolated *S*. *mutans* phage exhibits promising antimicrobial properties.

## Introduction

The microbiome of the human oral cavity is composed of numerous and diverse bacteria, archaea, eukaryotes and viruses [1–3]. Dental caries arise as a result of an ecological imbalance of metabolic activities in the stable oral microbiome. Dental caries is one of the most prevailing and persistent disease in the human population, despite the availability of various prophylactic options. *Streptococcus mutans* is a Gram-positive, coccus-shaped, non-motile and facultative anaerobic bacterium which is naturally present in the human mouth. It is an opportunistic pathogen and the principal etiological agent of dental decay in humans. *S*. *mutans* is able to adhere to the tooth surface in biofilm communities that contribute to dental plaque and favour the progression of dental disease [4, 5]. Within the dental plaque, *S*. *mutans* contributes greatly to the composition of the biofilm matrix, especially by producing abundant exopolysaccharides (EPS) [6]. *S*. *mutans* pathogenicity also results from its acidogenicity in the presence of dietary sucrose and its concomitant acid tolerance, both of which support changes in the ecology of the dental plaque by selecting for a cariogenic flora, increasing the probability of enamel demineralization and eventually caries formation [7]. When established as a biofilm, microbial communities are less sensitive to conventional antimicrobial interventions and, in any event, antibiotics are not favoured as a means of controlling or preventing caries.

Bacteriophage (phage) therapy is increasingly considered as a potential alternative to antibiotic treatments [8]. Phages are bacterial viruses that can attack and kill a target bacterium within minutes of infection. They are self-replicating and generally only target a narrow range of bacterial strains of the same species. Phages have been used in clinical settings for decades and are now accepted to be effective for the control of pathogenic bacteria in food [9]. Commercial phage cocktails, such as ListShield^TM^ and LISTEX^TM^ P100, are used against *Listeria monocytogenes* in the food industry and have obtained Generally Recognized As Safe (GRAS) status from the FDA [10]. While phages have been extensively used in some countries of Eastern Europe for clinical purposes, there are still no approved phage treatments in the Western world for clinical use [10]. However, promising research is being conducted on phages to treat bacterial pathogens such as *Pseudomonas aeruginosa* [11], enteroaggregative *Escherichia coli* [12], *Clostridium difficile* [13] and methicillin-resistant *Staphylococcus aureus* [14]. By discovering new phages effective against pathogenic bacteria, it is possible to develop alternative treatment methods to antibiotics. A few studies exist regarding the use of phages for curing dental infections with various pathogens such as *Enterococcus faecalis* [15], *Fusobacterium nucleatum* [16] and *P*. *aeruginosa* [17]. To our knowledge, no study has been dedicated to the use of phages to control *S*. *mutans* growth and biofilm formation. Relatively little is known about *S*. *mutans* phages. To date only two *S*. *mutans* phage genomes, M102 and M102AD, are available in public databases [18, 19]. Two other *S*. *mutans* phages, e10 and f1, have previously been isolated and tested for their host range and morphology, without being sequenced [20]. The aim of the current study was to isolate and characterize new *S*. *mutans* phages from saliva samples, and to test their efficacy in reducing *S*. *mutans* growth and biofilm formation for potential future application as antimicrobial agents.

## Materials and Methods

### Strains and culture conditions

*S*. *mutans* strains (Table 1) were grown overnight in BHI broth (Oxoid, Basingstoke, United Kingdom) at 37°C under aerobic conditions. All strains originated from the collection of Moorepark Food Research Centre (Ireland). Strain serotypes were checked using a PCR method described previously [21] (Table 1).

**Table 1:** *Streptococcus mutans* strains used in this study.

Strain No.	Origin	Serotype	Source and reference
DPC6143		e	
DPC6144		e	
DPC6145		c	
DPC6150		c	
DPC6151		c	
DPC6152		c	
DPC6153	Dental saliva isolate from University College Cork dental hospital	c	Culture collection of Moorepark Food Research Centre (Ireland) [48]
DPC6154		c	
DPC6155		c	
DPC6156		c	
DPC6157		c	
DPC6158		c	
DPC6159		e	
DPC6160		c	
DPC6161		c	
DPC6162	Carious dentine	e	Type strain NCTC10449
DPC6543	NA	c	University of Toronto

NA: not available

### Human subject enrolment

Subject recruitment and enrolment were approved by the Clinical Research Ethics Committee of the Cork Teaching Hospitals (protocol no. APC052). All subjects completed a questionnaire demonstrating their willingness to participate in the study. All subjects were healthy adults without known oral health problems. A minimum of 3 ml of saliva was collected in the morning before breakfast prior to any oral hygiene practices, and the saliva was analysed within 2 h after collection. A total of 85 samples were collected.

### Phage isolation from human saliva

One millilitre of saliva was centrifuged at 8000xg for 10 min, before being sterilised using 0.45 μm filters. Saliva filtrates were kept at 4°C. For each sample, 100 μl of saliva filtrate were mixed with 200 μl of bacterial overnight culture, and incubated for 20 min at 37°C. The indicator *S*. *mutans* strains used were DPC6143, DPC6144, DPC6145, DPC6150, DPC6151 and DPC6152 (Table 1). The filtrate-bacteria mixture was then added to 3 ml of soft BHI agar (0.5% agar w/v) containing 10 mM CaCl_2_, and overlaid on top of a BHI agar plate. Plates were incubated for a minimum of 24 h at 37°C or until plaques could be detected.

### Electron microscopic analysis

Phage lysates were purified on a caesium chloride gradient by ultracentrifugation, and were dialyzed against phage buffer (20 mM Tris-HCl [pH 7.2], 10 mM NaCl, 20 mM MgSO_4_) overnight at 4°C. Negative staining of phages and transmission electron microscopic analysis were as previously described [22].

### One-step growth curve

A one-step growth experiment was performed in triplicate to assess the burst size, and latency and rise phases of phage ɸAPCM01 using a method previously described [22] with the following modifications. Incubations were performed in BHI broth supplemented with 10 mM CaCl_2_, at 37°C. A multiplicity of infection (MOI) of 1 was used.

### Efficiency of lysogeny

Efficiency of lysogeny was assessed as previously described [23]. Briefly, 100 μl of 10^10^ pfu/ml phage lysate were spread onto BHI agar plates. An overnight culture of *S*. *mutans* DPC6143 strain was diluted by serial 1:10 dilutions. For 10^−4^ to 10^−7^ dilutions, 100 μl were mixed with 4 ml of sloppy BHI agar (0.5% agar) supplemented with 10 mM CaCl_2_, and overlaid onto phage seeded plates and phage-free control plates. The plates were incubated at 37°C for 24 h. Cfu numbers were count on countable plates, considering that the colonies growing on phage seeded plates were all lysogens. The percentage of efficiency of lysogeny was calculated as follows: (cfu on phage seeded plates / cfu on phage-free control plates) x 100. All experiments were performed in triplicate.

### Bacterial challenge and artificial saliva assays

Bacterial challenge and kill-curve assays were performed to determine the effect of different MOI’s of phage on *S*. *mutans* survival. An overnight culture of S. *mutans* DPC6143 strain was diluted in 2× GM17 broth (Oxoid) containing 20 mM CaCl_2_ to reach 10^4^ cfu/ml. The wells of a 96-well microplate were filled with 100 μl of the diluted culture. Serial 1:10 dilutions of the phage lysate were performed in phage buffer. The eight wells of each column of the 96-well microplate containing the diluted culture were filled with 100 μl of the same phage dilution. The range of MOI’s tested was between 2.5x10^-5^ and 2.5x10^2^. One column of the plate contained positive control wells with only 100 μl of the diluted *S*. *mutans* culture and 100 μl of phage buffer. Another column contained only 100 μl of 2× GM17 broth and 100 μl of phage buffer. The plate was incubated at 37°C for 18 h. Optical density (OD_600nm_) measures were taken and a Student’s t-test was performed to assess significance (GraphPad, Prism, version 5.03). Bacterial counts were performed in triplicate for each condition tested using the enumeration miniaturized method described previously [24].

Kill-curves were performed as described for the bacterial challenge assay, with OD_600nm_ measures recorded every 15 min using an MWGt Sirius HT plate reader (BIO-TEK® Instruments, USA).

The action of phages on *S*. *mutans* DPC6143 strain was also tested in artificial saliva [25] supplemented with 1% sucrose. An overnight culture of *S*. *mutans* DPC6143 strain was centrifuged at 8000×g for 10 min at room temperature. The supernatant was discarded and the pellet was resuspended in the same volume of 2x artificial saliva. The same method as for the bacterial challenge described above was applied to assess the action of phage against *S*. *mutans* in artificial saliva.

### Biofilm assays

Two 96-well plates were filled as followed. Each well was filled with 200 μl of BHI broth inoculated at 1% with an overnight culture of strain DPC6143. The plates were incubated at 37°C for 48 h to allow the biofilm to form. Broth containing planktonic cells was removed, being careful not to disturb the cells attached to the wells. 100 μl of 2× BHI broth containing 20 mM CaCl_2_ and 100 μl of lysate dilutions as describe above were added to each well. Phage lysate concentrations from 10^2^ to 10^9^ phage per well were tested, with each phage concentration being tested in 8 wells of the plate. The positive and negative controls were performed as described above. One plate was incubated at 37°C for 24 h and the other at 37°C for 48 h. After incubation, the wells were emptied carefully and gently washed with phosphate buffered saline. A colorimetric assay using XTT and menadione as previously described [26] was performed to assess the metabolic activity of *S*. *mutans* biofilm after phage treatment. Bacterial counts were performed in triplicate for each condition tested. Briefly, biofilms were detached from the wells by thorough mixing by pipetting with 200 μμl maximum recovery diluent (Oxoid), and counts were carried out following the enumeration miniaturized method [24].

### DNA extraction and genome sequencing

DNA was extracted from the CsCl purified fractions [22]. Briefly, 400 μl of the CsCl purified fraction were treated with 6 U Ambion® TURBO^TM^ DNase (Life technologies, USA). After DNase treatment, 4 μl of proteinase K (20mg/ml) and 30 μl of 10% SDS were added and followed by an incubation step of 1h at 56°C. Another incubation step of 10 min at 65°C followed after the addition of 70 μl 5 M NaCl and 100 μl of phage lysis buffer (4.5 M guanidine thiocyanate, 44 mM sodium citrate [pH 7.0], 1% sarkosyl, 72 μl 2-mercaptoethanol). DNA was then extracted and purified using phenol:chloroform:isoamyl alcohol (25:24:1, Sigma-Aldrich, Saint-Louis, USA), and precipitated with ice-cold ethanol. DNA samples were sent to GATC (Konstanz, Germany) for whole phage genome sequencing using an Illumina HiSeq 2500 sequencer with 2x100 bp read length. The reads generated by the Illumina instrument were assembled at GATC.

### *In silico* genome analysis

Protein-encoding open reading frames (ORFs) were predicted using Glimmer [27] and the RAST server [28]. Initial functional annotation of the ORFs and percentage amino acid identities were determined using BLASTP [29]. Phylogenetic trees were constructed using streptococcal endolysin amino acid sequences which gave the highest possible identity percentage with endolysins in phage ɸAPCM01 genome. The evolutionary history was inferred by using the Maximum Likelihood method based on the JTT matrix-based model [30] in MEGA 6 [31]. The bootstrap consensus tree inferred from 1000 replicates was taken to represent the evolutionary history of the taxa analysed [32]. Branches corresponding to partitions reproduced in less than 50% bootstrap replicates were collapsed. Initial tree(s) for the heuristic search were obtained by applying the Neighbor-Joining method to a matrix of pairwise distances estimated using a JTT model.

### Accession number

The complete genome sequence of ɸAPCM01 has been deposited in GenBank under accession number KR153145.

## Results

### Morphology, host range, population dynamics, and lysogeny

**Fig 1:**
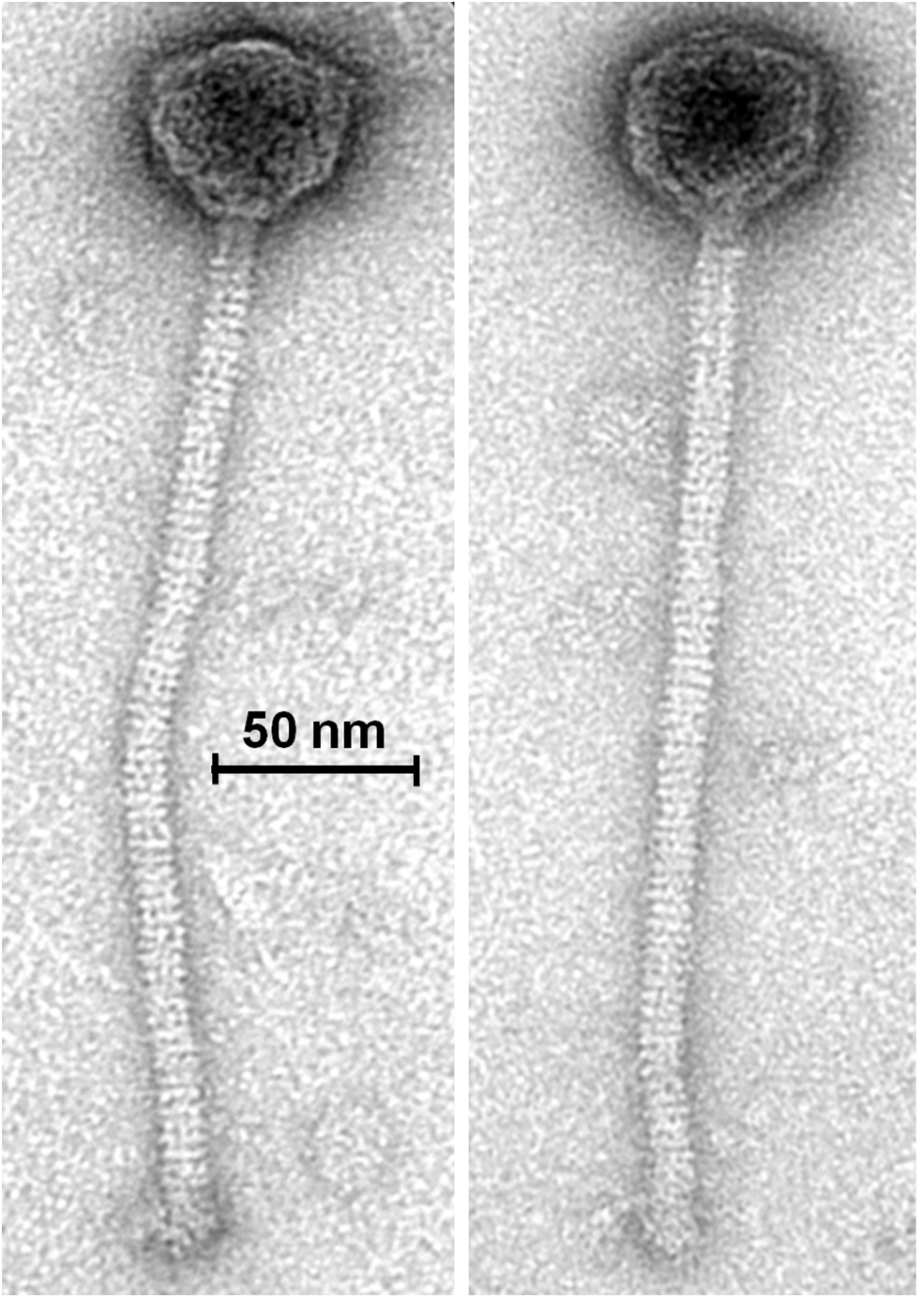
Transmission electron micrograph of phage ɸAPCM01, stained with uranyl acetate.

Given the potential of phage therapy, a screening of 85 saliva samples was performed to isolate *S*. *mutans* phage. A single *S*. *mutans* phage, ɸAPCM01, was isolated from one saliva sample. ɸAPCM01 belongs to the (small-isometric headed) *Siphoviridae* family with B1 morphology as shown by electron microscopy (Fig 1). The head diameter was 54.6 ± 1.0 nm (n = 7) and the length of the non-contractile and flexible tail was 278.0 ± 9.7 nm (n = 7). Small baseplate structures (width: 16.1 ± 0.8 nm [n = 7]) without further appendices or fibers were visible at the distal end of the tails (tail width: 11.5 ± 0.4 nm [n = 7]). Of the 17 *S*. *mutans* strains tested ɸAPCM01 targeted only strain DPC6143.

**Fig 2:**
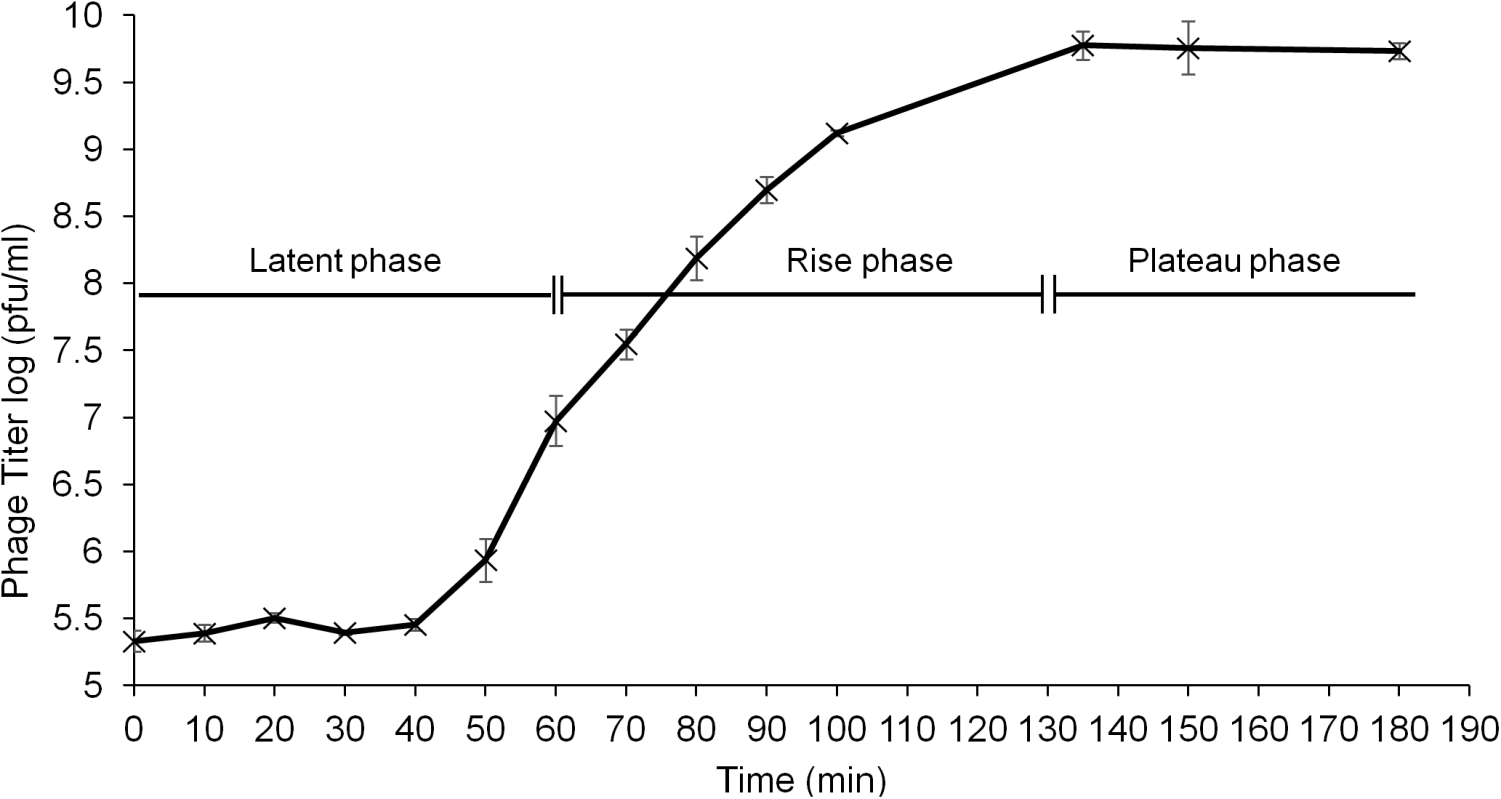
One-step growth curve of phage ɸAPCM01 with *S*. *mutans* strain DPC6143 in BHI broth at 37°C. Three independent experiments were carried out. Error-bars indicate standard deviation.

A one-step growth curve was performed to assess the population dynamics of ɸAPCM01 in the presence of *S*. *mutans* strain DPC6143. ɸAPCM01 had a latent period of 60 min and a rise phase of 60 min before reaching the plateau phase (Fig 2). The burst size was calculated as 44.2 ± 9.8 phage particles.

Lysogeny of phage ɸAPCM01 was assessed against *S*. *mutans* strain DPC6143. The numbers of bacteria on phage seeded plates and on phage-free control plates were 5.7 ± 0.2 and 9.3 ± 0.09 log cfu/ml, respectively. This gave a mean efficiency of lysogeny of 0.027 ± 0.007%.

### Bacterial challenge

**Fig 3:**
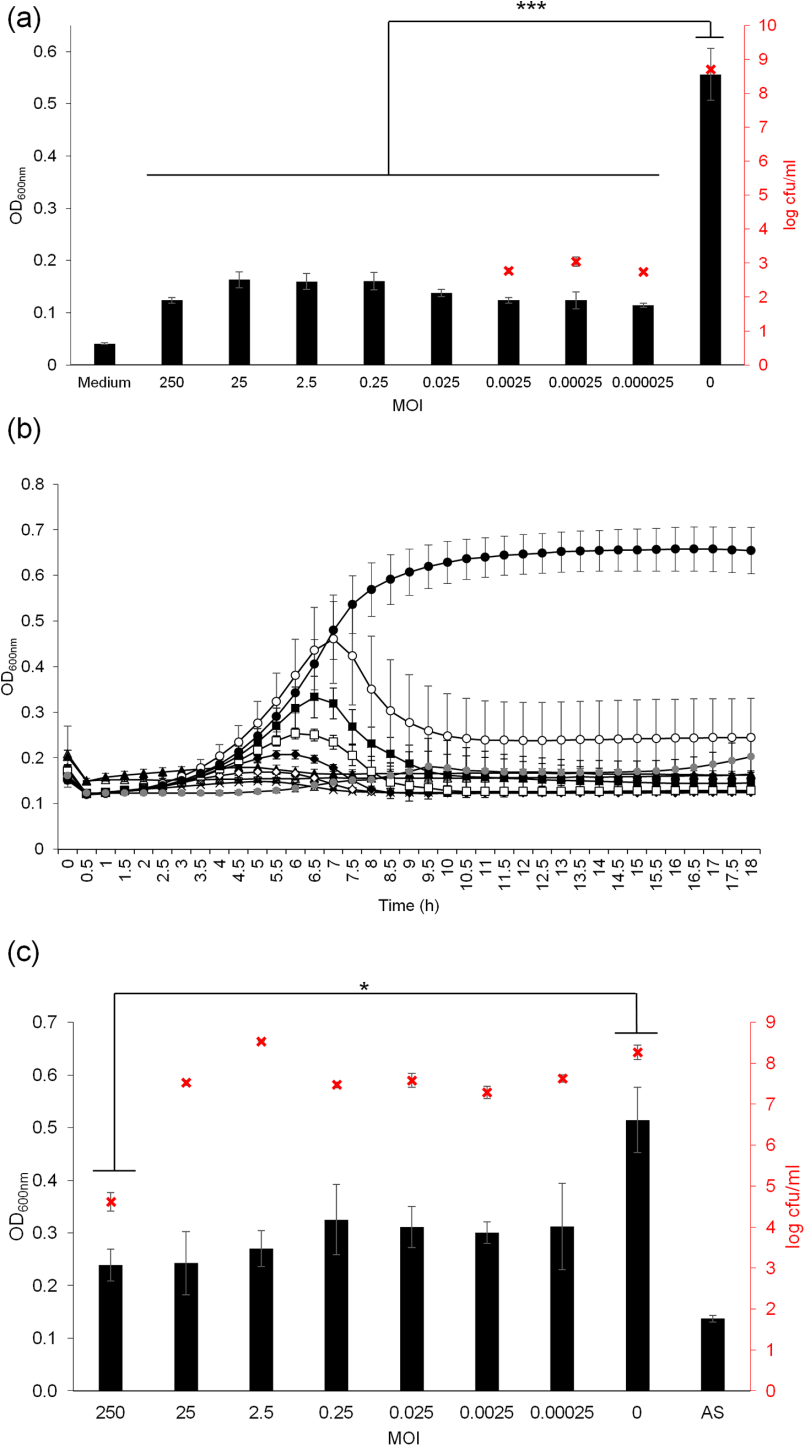
Effect of phage ɸAPCM01 on a growing culture of *S*. *mutans* DPC6143. (a) Phage activity was assessed by OD_600nm_ measures after 18 h of contact in BHI broth at 37°C. Experiments were performed in a 96-well microplate, and each condition was tested in 8 wells of the microplate. Enumerations were performed in triplicate for each tested MOI (**x**), with a detection threshold of 20 cfu/ml. (b) Killing curves were assessed by OD_600nm_ measures every 15 min for 18 h at MOI of 2.5x10^-5^ (○), 2.5x 10^−4^ (■), 2.5x 10^−3^ (□), 2.5x 10^−2^ (◆), 0.25 (◇), 2.5 (☓), 25 (▲), 250 (△), no phage (●), and sterile medium (•). (c) Phage activity was assessed by OD_600nm_ measures and by bacterial counts (**x**) performed in triplicate after 18 h of contact in artificial saliva. MOI: multiplicity of infection; AS: sterile artificial saliva. Error-bars indicate standard deviation. ***p<0.001.

The ability of ɸAPCM01 to reduce or prevent the growth of *S*. *mutans* DPC6143 was assessed after 18 h of contact with the phage at MOI’s ranging from 2.5x10^-5^ to 250 (Fig 3A). A significant decrease in OD_600nm_ of at least 2-fold between the control culture and the cultures with added phages was observed for all the tested MOI’s (p-value<0.001). This indicates that the phage was efficient even at a low MOI. This was confirmed by a decrease of at least 5.6 log cfu/ml between the control culture and the cultures with added phages at MOI’s smaller than 2.5x10^-3^ (Fig 3A). At MOI’s higher than 2.5x10^-2^ no colonies could be detected (detection threshold of 20 cfu/ml). ɸAPCM01 prevented the growth of *S*. *mutans* DPC6143 at MOI’s higher than 2.5x10^-2^ or reduced its growth at lower MOI’s.

Kill curves confirmed this result and indicated that *S*. *mutans* DPC6143 was inhibited by ɸAPCM01 at an early stage of growth (Fig 3B). At MOI’s higher than 10^−2^, no growth occurred as OD_600nm_ values remained the same as the sterile medium OD_600nm_ values during the entire incubation period. At MOI’s equal to, or less than, 10^−3^, some growth initially occurred but the OD declined after 7 to 10 hours of incubation.

The lytic activity of ɸAPCM01 against *S*. *mutans* DPC6143 strain was also assessed in artificial saliva (Fig 3C). The OD_600nm_ decreased by at least 1.5 fold compared to the control culture without phage. Bacterial counts revealed that a significant reduction of about 3.6 log cfu/ml (p-value<0.05) was only observed between the highest tested MOI and the control. Even if the decrease was less dramatic in artificial saliva than in culture broth, the phage could still reduce *S*. *mutans* DPC6143 growth in artificial saliva.

### Action of phage against biofilms

**Fig 4:**
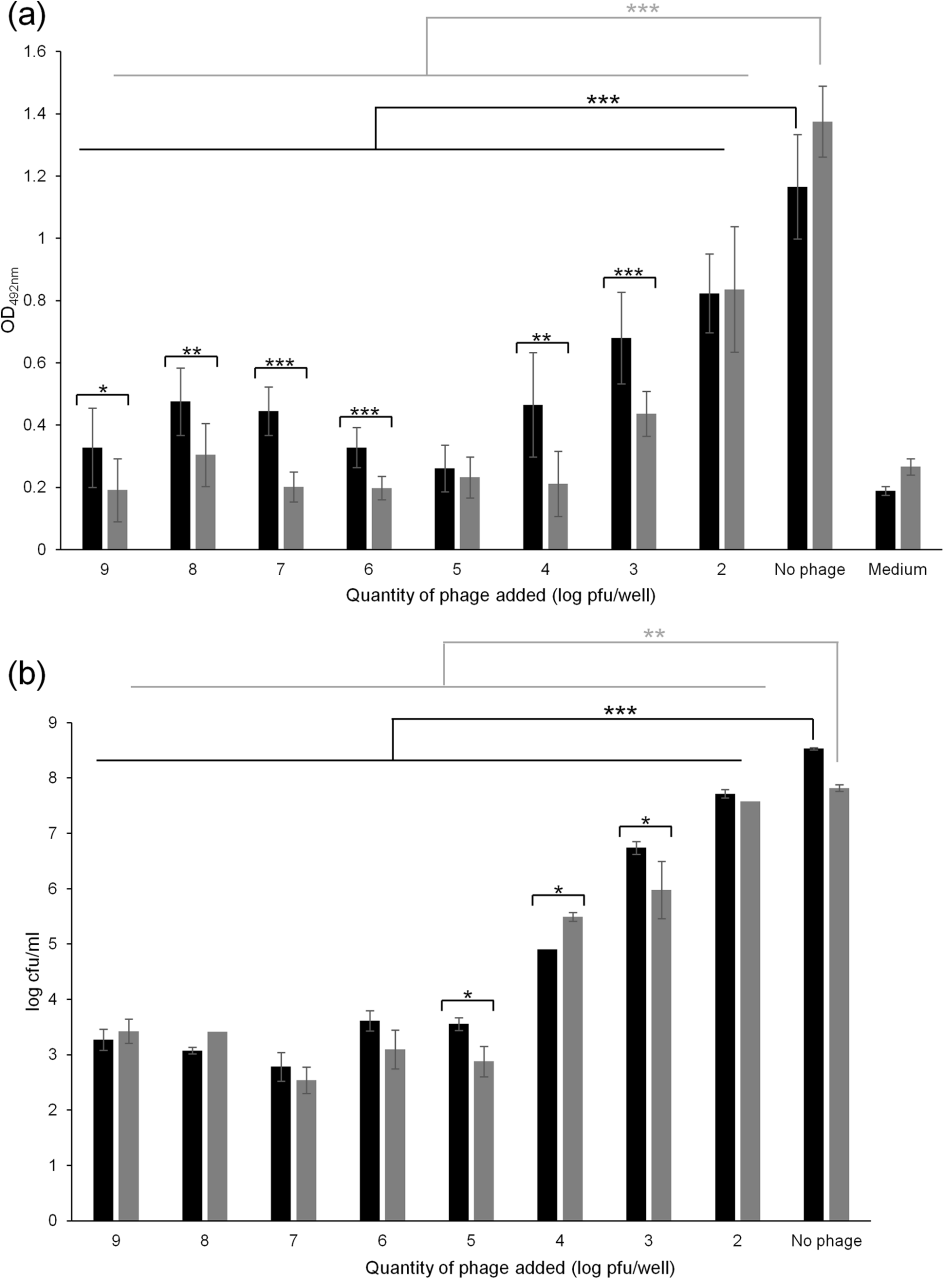
Effect of phage ɸAPCM01 on a 48 h-biofilm formed by *S*. *mutans* DPC6143, after 24 h (■) and 48 h (■) of contact between phage and biofilm. (a) Biofilm metabolic activity was assessed by OD_492nm_ measures after treatment with XTT supplemented with menadione. Experiments were performed in 96-well microplates, and each condition was tested in 8 wells of the microplate. (b) Bacterial counts in biofilms were performed in triplicate after contact with the phage. *p<0.05; **p<0.01; ***p<0.001. Error-bars indicate standard deviation.

Phage ɸAPCM01 was tested against 48 h-attached *S*. *mutans* DPC6143 cells in 96-well plates (Fig 4). After phage treatment, the metabolic activity of the biofilm was assessed using XTT assays and the numbers of live cells were quantified by bacterial counts. With initial doses equal to or higher than 10^2^ pfu/well, ɸAPCM01 significantly reduced the biofilm activity within 24 h of contact (p-value<0.001, Fig 4A). At doses ranging from 10^5^ to 10^9^ pfu/well, phage ɸAPCM01 completely inhibited the biofilm metabolic activity as shown by OD_492nm_ values close to 0.2, the OD value of the medium alone. At phage doses between 10^2^ and 10^4^ pfu/well, OD_492nm_ values were lower compared to the control but high enough to indicate residual metabolic activity of *S*. *mutans*. At these doses, phage ɸAPCM01 could reduce the biofilm but not completely disrupt it. Bacterial counts confirmed the reduction of the biofilm biomass due to phage action after 24 h of contact (Fig 4B). A difference of at least 5 log cfu/ml was noticeable between the control biofilm and the biofilm in the presence of phage doses higher than 10^5^ pfu/well (Fig 4B). A difference of 1, 1.8 and 3.5 log cfu/ml was measured between the control and the biofilm at phage doses of 10^2^, 10^3^ and 10^4^ pfu/well, respectively (Fig 4B).

After 48 h of contact with phage ɸAPCM01, the biofilm metabolic activity measured by XTT was significantly reduced at phage doses of 10^3^, 10^4^ and 10^6^ to 10^9^ pfu/well compared to the 24 h-incubation step (p-value<0.05) (Fig 4A). For these doses, OD_492nm_ was close to the OD_492nm_ of the medium. At phage doses of 10^2^ and 10^5^ pfu/well, no significant changes in the OD_492nm_ values were observed after 48 h of contact with phage compared to the 24 h-incubation step, indicating the absence of biofilm development. At phage doses ranging from 10^6^ to 10^9^ pfu/well, no significant differences in the number of bacterial cells between 24 h and 48 h of contact with the phage were noticeable (p-value>0.05, Fig 4B). This indicated that phage ɸAPCM01 is able to control the growth of the *S*. *mutans* biofilm. An increase of 0.6 log cfu/ml at a phage dose of 10^4^ pfu/well was visible (p<0.05) after 48 h. A decrease of 0.7 log cfu/ml at phage doses of 10^5^ and 10^3^ pfu/well was also noticeable (Fig 4B).

### Genome features and comparison with *S*. *mutans* phage M102 and M102AD genomes

**Fig 5:**
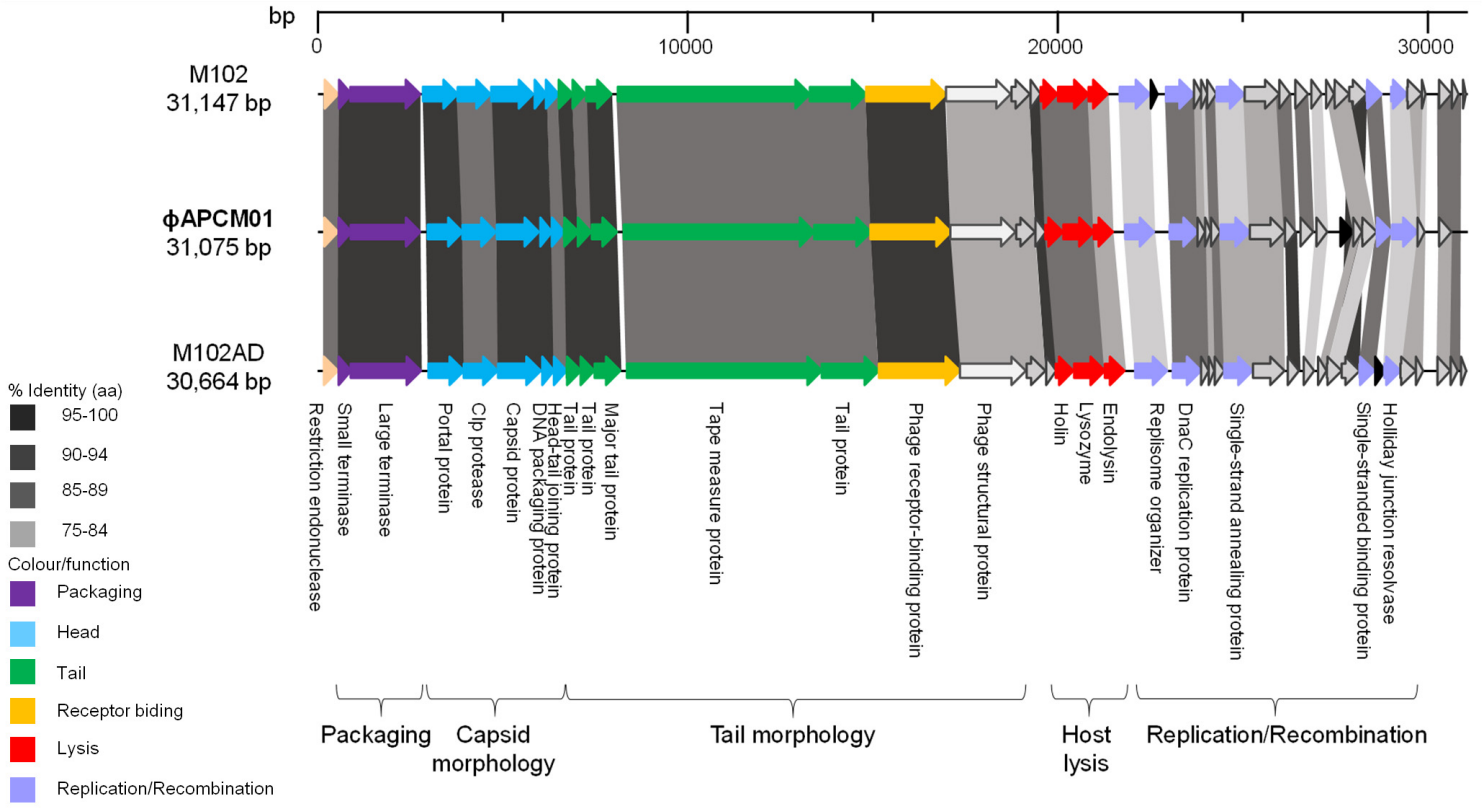
Genomic organization of ɸAPCM01 compared with that of phages M102 and M102AD. Each arrow represents an ORF, with the colour representing the putative function of the encoded protein indicated on the right. Percent amino acid identity between adjacent genomes is colour coded as outlined to the left.

The complete genome size of phage ɸAPCM01 is 31,075 bp with a G-C content of 39%. A total of 37 ORFs were identified on the same strand and 23 could be assigned a putative function. No tRNA encoding regions were found in ɸAPCM01’s genome. The ɸAPCM01 genome compares closely with two other *S*. *mutans* phage genomes and is organized into the following functional modules: DNA packaging, morphogenesis, lysis, and DNA replication and recombination (Fig 5). ɸAPCM01 shares 85% identity with M102 and M102AD at the nucleotide level. However, some discrepancies in the number of ORFs exists between the three phages. ORF31, encoding a protein of unknown function, is only present in the ɸAPCM01 genome while four ORFs which are present in the other phage genomes (M102 ORF34, ORF37, ORF40, ORF41) are missing in ɸAPCM01 (Fig 5). All these ORFs are located in the replication module and encode proteins of unknown function.

The maximum identity at the amino acid level is 99% (ORF29, small sub-unit terminase) (Table 2). Twenty and 22 ORFs out of 37 (54% and 59% of ORFs, respectively) from ɸAPCM01 share more than 90% identity at the amino acid level with M102AD and M102, respectively. ORF29 is also close to ORF30 of phage M102 with 93% identity at the amino acid level; this ORF is absent from M102AD genome.

**Table 2:** ORFs of phage ɸAPCM01: putative functions and identity comparison to phages M102 and M102AD ORFs.

							M102AD		M102	
ORF	Start position	Stop position	Size (aa)	Molecular mass (kDa)	pI	Putative function	Best match (% amino acid identity)	E-value	Best match (% amino acid identity)	E-value
ORF1	179	547	122	14.4	9.62	Endonuclease	ORF1 M102AD (91.8%, 122/122)	6.0E-88	ORF1 M102 (92.62%, 122/122)	3.0E-88
ORF2	563	907	114	13.3	5.87	Terminase, small subunit	ORF2 M102AD (99.11%, 112/114)	6.0E-83	ORF2 M102 (99.11%, 112/114)	6.0E-83
ORF3	894	2768	624	71.4	5.25	Terminase, large subunit	ORF3 M102AD (95.67%, 624/624)	0.0E+00	ORF3 M102 (95.51%, 624/624)	0.0E+00
ORF4	2970	3914	314	35.3	5.58	Portal protein	ORF4 M102AD (94.9%, 314/314)	0.0E+00	ORF4 M102 (97.13%, 314/314)	0.0E+00
ORF5	3911	4813	300	32.4	4.18	Clp protease-like protein	ORF5 M102AD (93.33%, 300/300)	0.0E+00	ORF5 M102 (92.33%, 300/300)	0.0E+00
ORF6	4834	5970	378	41.0	5.01	Capsid protein	ORF6 M102AD (95.77%, 378/378)	0.0E+00	ORF6 M102 (97.62%, 378/378)	0.0E+00
ORF7	6013	6324	103	11.7	4.36	DNA packaging	ORF7 M102AD (96.12%, 103/103)	2.0E-72	ORF7 M102 (97.09%, 103/103)	1.0E-73
ORF8	6321	6665	114	13.3	9.17	Head-tail joining protein	ORF8 M102AD (92.98%, 114/114)	3.0E-78	ORF8 M102 (92.11%, 114/114)	2.0E-77
ORF9	6658	7050	130	15.0	6.44	Tail protein	ORF9 M102AD (96.92%, 130/130)	5.0E-95	ORF9 M102 (95.35%, 129/130)	3.0E-93
ORF10	7034	7390	118	13.3	4.35	Tail protein	ORF10 M102AD (97.46%, 118/118)	2.0E-83	ORF10 M102 (94.07%, 118/118)	2.0E-81
ORF11	7408	8085	225	24.0	5.55	Major tail protein	ORF11 M102AD (95.09%, 224/225)	9.0E-154	ORF11 M102 (97.32%, 224/225)	3.0E-164
ORF12	8263	13419	1718	187.1	9.73	Tape-measure protein	ORF12 M102AD (90.14%, 1734/1718)	0.0E+00	ORF12 M102 (90.83%, 1734/1718)	0.0E+00
ORF13	13419	14945	508	57.4	5.73	Tail protein	ORF13 M102AD (92.72%, 508/508)	0.0E+00	ORF13 M102 (92.52%, 508/508)	0.0E+00
ORF14	14942	17110	722	81.3	5.01	Receptor binding protein	ORF14 M102AD (95.26%, 718/722)	0.0E+00	ORF14 M102 (94.99%, 718/722)	0.0E+00
ORF15	17100	18857	585	64.7	5.22	Structural protein	ORF15 M102AD (87.69%, 585/585)	0.0E+00	ORF15 M102 (87.52%, 585/585)	0.0E+00
ORF16	18880	19383	167	18.8	5.09	unknown	ORF16 M102AD (88.62%, 167/167)	3.0E-113	ORF16 M102 (88.02%, 167/167)	2.0E-112
ORF17	19401	19673	90	10.6	8.85	unknown	ORF17 M102AD (98.89%, 90/90)	3.0E-63	ORF17 M102 (98.89%, 90/90)	3.0E-63
ORF18	19670	20137	155	16.8	5.89	Holin	ORF18 M102AD (89.68%, 155/155)	1.0E-88	ORF18 M102 (89.68%, 155/155)	1.0E-88
ORF19	20153	20971	272	29.7	5.16	Lysozyme	ORF19 M102AD (93.77%, 273/272)	0.0E+00	ORF19 M102 (93.77%, 273/272)	0.0E+00
ORF20	20971	21486	171	18.7	6.05	Endolysin	ORF20 M102AD (88.3%, 171/171)	2.0E-114	ORF20 M102 (88.3%, 171/171)	2.0E-114
ORF21	21822	22601	259	30.0	8.89	Replisome organizer	ORF21 M102AD (76.84%, 272/259)	6.0E-160	ORF21 M102 (77.21%, 272/259)	7.0E-161
ORF22	23023	23781	252	29.3	8.68	DNAc replication protein	ORF22 M102AD (90.87%, 252/252)	2.0E-164	ORF23 M102 (89.68%, 252/252)	3.0E-164
ORF23	23781	23984	67	8.0	9.99	unknown	ORF23 M102AD (88.06%, 67/67)	2.0E-41	ORF24 M102 (92.54%, 67/67)	4.0E-43
ORF24	23981	24133	50	6.1	9.87	unknown	ORF24 M102AD (82%, 50/50)	1.0E-29	ORF25 M102 (88%, 50/50)	1.0E-31
ORF25	24144	24392	82	9.2	4.15	unknown	ORF25 M102AD (91.46%, 82/82)	1.0E-54	ORF26 M102 (91.46%, 82/82)	1.0E-54
ORF26	24402	25190	262	29.9	5.36	Single-strand annealing protein	ORF26 M102AD (83.4%, 235/262)	3.0E-145	ORF27 M102 (85.53%, 235/262)	5.0E-149
ORF27	25205	26134	309	35.6	8.12	unknown	ORF27 M102AD (89.43%, 265/309)	7.0E-159	ORF28 M102 (87.01%, 308/309)	0.0E+00
ORF28	26135	26431	98	11.4	10.05	unknown	ORF28 M102AD (91.84%, 98/98)	4.0E-64	ORF29 M102 (95.92%, 98/98)	2.0E-66
ORF29	26561	26905	114	13.6	9.66	unknown			ORF30 M102 (93.86%, 114/114)	2.0E-75
ORF30	26993	27277	94	10.9	9.87	unknown	ORF29 M102AD (80.43%, 92/94)	2.0E-51	ORF31 M102 (73.12%, 93/94)	4.0E-48
ORF31	27639	27992	117	13.5	5.02	unknown				
ORF32	27985	28212	75	9.1	9.91	unknown	ORF30 M102AD (89.19%, 74/75)	4.0E-47	ORF32 M102 (89.33%, 75/75)	2.0E-48
ORF33	28218	28613	131	15.3	9.89	unknown	ORF31 M102AD (80.92%, 131/131)		ORF33 M102 (87.79%, 131/131)	1.0E-87
ORF34	28620	29015	131	15.2	7.95	Single-stranded DNA-binding protein	ORF32 M102AD (93.13%, 131/131)	4.0E-90	ORF35 M102 (91.6%, 131/131)	1.0E-88
ORF35	29030	29722	230	27.2	9.45	Holliday junction resolvase	ORF35 M102AD (75.71%, 140/230)	3.0E-80	ORF36 M102 (75.35%, 142/230)	5.0E-81
ORF36	29715	29912	65	7.2	7.78	unknown	ORF37 M102AD (84.62%, 65/65)	3.0E-38	ORF38 M102 (75%, 32/65)	1.0E-14
ORF37	30297	30641	114	13.3	5.05	unknown	ORF38 M102AD (89.47%, 114/114)	2.0E-71	ORF39 M102 (91.23%, 114/114)	2.0E-74

### Comparison of phage ɸAPCM01 endolysins to other streptococcal phage endolysins

**Fig 6:**
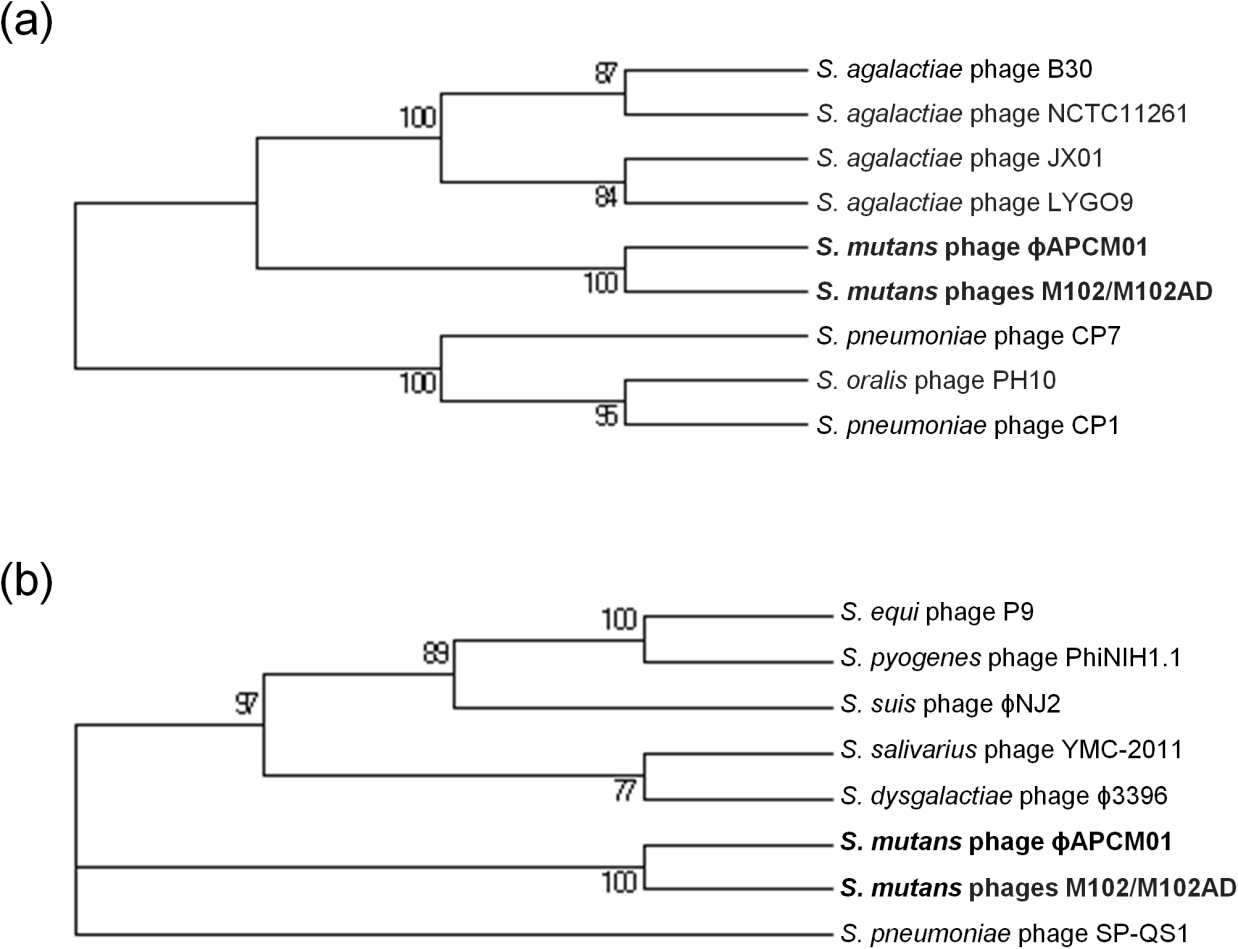
Molecular phylogenetic analysis by Maximum Likelihood method of endolysins in *S*. *mutans* phage ɸAPCM01. Comparison of ORF19 (a) and ORF20 (b) of ɸAPCM01 to other endolysins in streptococcal phages. Numbers indicate branches support based on 1000 bootstrap replications.

Phages M102 and M102AD endolysins (ORF19 and ORF20) were shown to be 100% identical and to share similarity with other streptococcal endolysins [18]. Interestingly, ORF19 and ORF20 of ɸAPCM01 are not identical to the corresponding ORFs of phages M102 and M102AD, and share 93.7% and 88.3% identity at the amino acid level, respectively (Table 2). *S*. *mutans* phage endolysins were then compared to other streptococcal endolysins which gave the closest BLAST identity values, and a phylogeny of these phages was established based on ORF19 (Fig 6A) and ORF20 (Fig 6B). In both cases, *S*. *mutans* phages constitute a distinct group from the other *Streptococcus* species. Based on ORF19 phylogeny, *S*. *mutans* phages are closer to *S*. *agalactiae* phage than to other streptococcal phages (Fig 6A). The phylogeny based on ORF20 could not relate *S*. *mutans* phages to any particular *Streptococcus* species (Fig 6B).

## Discussion

*S*. *mutans* is the leading cause of dental caries worldwide and is considered to be the most cariogenic of all of the oral streptococci. Very little is known about *S*. *mutans* lytic phages. To our knowledge only two *S*. *mutans* phages, M102 and M102AD, have been sequenced to date [18, 19]. Phage M102 was isolated in 1988 in France [33], and M102AD came from a M102 batch kept at the University of Maryland which proved to be genetically different from the original phage M102 after genome comparison of the two phages [18]. Two other *S*. *mutans* phages have also been partially characterized in the University of Maryland, phages e10 and f1 [20]. A paper from the late 70’s also relates the presence of prophage inducible with mitomycin C in the genome of some *S*. *mutans* strains [34]. The scarcity of data on *S*. *mutans* phages is probably due to the difficulty to isolate phages from oral cavity samples. In the current study, only one phage, ɸAPCM01, was isolated from screening 85 saliva samples. Two other studies aiming at isolating phages against oral pathogens failed to isolate *S*. *mutans* phages [35, 36]. This could possibly be due to the low frequency of these phages in nature or, more probably, to their narrow host range. ɸAPCM01 isolated in the present study only targeted *S*. *mutans* strain DPC6143 out of the 17 tested strains. The fact ɸAPCM01 targets this strain is of importance as this strain is serotype e, which is the second more present *S*. *mutans* serotype in the oral cavity after serotype c [37]. Phages M102, M102AD, e10 and f1 also proved to have a narrow host-range. In addition, these phages appear to be serotype-specific, although not all strains of the same serotype are sensitive to a given phage [18, 20], which was the case for ɸAPCM01. Indeed, the strain targeted by ɸAPCM01 was serotype e, and other serotype e strains tested in the present study were not sensitive to the phage. The sensitivity to phage ɸAPCM01 of other *S*. *mutans* strains, especially of serotype c and e, should be examined to further elucidate this aspect. Adsorption of M102 phage to *S*. *mutans* cells depends on the type of the glucose side chain of the rhamnose-glucose-polysaccharides which constitute the receptor for phage M102 [38]. Moreover CRISPR sequences matching the *S*. *mutans* phage M102 genome were detected in some *S*. *mutans* strains, indicating a bacterial resistance mechanism to this particular type of phage [39, 40]. Other phage resistance systems also exist and are frequently discovered, like the recent bacteriophage exclusion system BREX [41]. All these factors may make it more difficult to find appropriate *S*. *mutans* strains with which to isolate virulent phages.

Like the four other reported *S*. *mutans* phages, ɸAPCM01 belongs to the *Siphoviridae* family. Its head and tail dimensions are in accordance with that of the other *S*. *mutans* phages [18, 20]. The ɸAPCM01 genome (31,075 bp) is slightly smaller than that of phage M102 (31,147 bp) [19] and bigger than that of phage M102AD (30,664 bp) [18]. ɸAPCM01 displays 85% nucleotide identity with M102 and M102AD and differs in that it has a reduced number of ORFs, albeit with an additional ORF31 specific to its genome. None of its predicted proteins are identical to those of the two previously sequenced phages. The fact that this new *S*. *mutans* phage is so closely related to existing *S*. *mutans* phage begs the question of the origin and evolution of these phages, and of the population structure. As observed for phage M102AD, it seems that the evolution of phage ɸAPCM01 genome is due to point mutations, and to the deletion and acquisition of genes [18], with no certainty regarding the frequency and nature of events causing those modifications. As the number of available *S*. *mutans* phage genomes is so limited a phylogenetic explanation of these events is not yet possible. We attempted to compare *S*. *mutans* phages to other phages by comparing their endolysins (ORF19 and ORF20). The ɸAPCM01 endolysin encoded by ORF19 is related to endolysins from different *S*. *agalactiae* phages. This is congruent with the observation previously made for phage M102AD where the endolysin shares 49% of its amino acids with *S*. *agalactiae* phage B30 endolysin [18]. An increased effort in isolating and sequencing more *S*. *mutans* phages will be required for a better understanding of the phage population structure, taxonomy and evolution [42]. The closeness of these three phage genomes isolated in different countries and almost 30 years apart strongly suggests an evolution from a common ancestor, and the existence of a cohesive population of *S*. *mutans* phages where genetic modification events occur at a very low rate.

Phage therapy is increasingly considered as a viable alternative for the treatment and control of pathogenic bacteria [43]. To date, the antimicrobial potential of *S*. *mutans* phage has not been extensively studied. In this study we tested the ability of ɸAPCM01 to reduce *S*. *mutans* growth and biofilm formation. Phage ɸAPCM01 was proven to be highly lytic with a burst size of ~44 against *S*. *mutans* strain DPC6143. Phage ɸAPCM01 also showed a lack of lysogenic properties with an efficiency of lysogeny of less than 0.03%, confirmed by the absence of a lysogeny module in its genome. If the colonies growing on phage-seeded plates were considered as bacteriophage-insensitive mutants (BIMs) [44], the lysogeny assay could also indicate that the frequency of BIMs is extremely low (less than 0.03%) in the presence of ɸAPCM01. These criteria make phage ɸAPCM01 a suitable candidate for phage therapy. ɸAPCM01 efficiently reduced the growth of *S*. *mutans* by at least 5 log cfu/ml in laboratory broth, and by at least 3 log cfu/ml in artificial saliva supplemented with sucrose. The latter ability is of great interest as it is closer to the real conditions of contact between *S*. *mutans* and its phages. It has been shown that the combination of saliva, sucrose water and nutrients had synergistic effects on *S*. *mutans* growth and long-term colonization [45]. Based on the results of the current study, the addition of phages could help in reducing the colonization of teeth surface by *S*. *mutans*. This is particularly true in regard to the ability of ɸAPCM01 to reduce *S*. *mutans* biofilm in a model system after a minimum 24 h of contact. The metabolic activity of the biofilm was significantly reduced, and the number of live cells decreased by at least 5 log cfu/ml with phage doses of at least 10^5^ pfu/well. After 48 h of contact with phage, the reduction of the metabolic activity of the biofilm did not systematically come with a reduction in the numbers of cells, which remained essentially stable. This indicates the prolonged control of the biofilm by the phage in time. The decrease in the metabolic activity of the biofilm could be due to the exhaustion of nutrients in the medium even if no significant differences in the control were visible after 48 h. To accurately explain the metabolic activity of the biofilm multiparametric measurements would need to be used as previously performed for *S*. *mutans* biofilms [46]. The reduction of *S*. *mutans* growth and biofilm formation has successfully been tested by the use of other antimicrobial agents such as essential oils and bioactive fractions [47], bacteriocins [48], commensal bacteria [49] and probiotic bacteria [50]. Combining these approaches with the use of phage ɸAPCM01 could compensate for the narrow host range of this phage at the current stage of investigation, and thus could increase the chances to prevent the development of *S*. *mutans* biofilm and caries formation. The combination of ɸAPCM01 with other *S*. *mutans* phages is also of utmost importance as phage cocktails have proven to be more efficient [51] because they limit the risk of bacterial adaptation and the emergence of resistance [52]. The combination of different phages would also permit to extend the host range of the phage cocktail and thus would improve its efficiency.

In conclusion, while the narrow host range is a significant disadvantage, the newly isolated phage ɸAPCM01 has promising antimicrobial properties, such as the ability to reduce *S*. *mutans* growth and biofilm formation. Its use in combination with other phages and antimicrobial agents can now be considered for future potential clinical use.

## References

[1] Nasidze I, Li J, Quinque D, Tang K, Stoneking M. Global diversity in the human salivary microbiome. Genome Res. 2009; 19(4): 636–43. 10.1101/gr.084616.108 . 19251737 PMC2665782

[2] Pride DT, Salzman J, Haynes M, Rohwer F, Davis-Long C, White RA 3rd, et al Evidence of a robust resident bacteriophage population revealed through analysis of the human salivary virome. ISME journal. 2012; 6(5): 915–26. 10.1038/ismej.2011.169 MEDLINE:. 22158393 PMC3329113

[3] Ghannoum MA, Jurevic RJ, Mukherjee PK, Cui F, Sikaroodi M, Naqvi A, et al Characterization of the oral fungal microbiome (mycobiome) in healthy individuals. PLoS pathog. 2010; 6(1): e1000713 10.1371/journal.ppat.1000713 20072605 PMC2795202

[4] Motegi M, Takagi Y, Yonezawa H, Hanada N, Terajima J, Watanabe H, et al Assessment of genes associated with *Streptococcus mutans* biofilm morphology. Appl Environ Microbiol. 2006; 72(9): 6277–87. 10.1128/aem.00614-06 16957255 PMC1563623

[5] Ahn SJ, Ahn SJ, Wen ZT, Brady LJ, Burne RA. Characteristics of biofilm formation by *Streptococcus mutans* in the presence of saliva. Infect Immun. 2008; 76(9): 4259–68. 10.1128/iai.00422-08 . 18625741 PMC2519434

[6] Klein MI, Hwang G, Santos PHS, Campanella OH, Koo H. *Streptococcus mutans*-derived extracellular matrix in cariogenic oral biofilms. Frontiers in cellular and infection microbiology. 2015; 5 10.3389/fcimb.2015.00010 . PMC4327733 25763359

[7] Banas JA. Virulence properties of *Streptococcus mutans* . Front Biosci. 2004; 9: 1267–77. . 14977543 10.2741/1305

[8] Parasion S, Kwiatek M, Gryko R, Mizak L, Malm A. Bacteriophages as an alternative strategy for fighting biofilm development. Polish journal of microbiology / Polskie Towarzystwo Mikrobiologow = The Polish Society of Microbiologists. 2014; 63(2): 137–45. . 25115107

[9] Endersen L, O'Mahoney J, Hill C, Ross RP, McAuliffe O, Coffey A. Phage therapy in the food industry. Annu Rev Food Sci Technol. 2014; 5(1): 327–49. 10.1146/annurev-food-030713-092415 24422588

[10] Lu TK, Koeris MS. The next generation of bacteriophage therapy. Curr Opin Microbiol. 2011; 14(5): 524–31. 10.1016/j.mib.2011.07.028 . 21868281

[11] Alemayehu D, Casey PG, McAuliffe O, Guinane CM, Martin JG, Shanahan F, et al Bacteriophages ϕMR299-2 and ϕNH-4 can eliminate *Pseudomonas aeruginosa* in the murine lung and on cystic fibrosis lung airway cells. mBio. 2012; 3(2): e00029–12. 10.1128/mBio.00029-12 . 22396480 PMC3302570

[12] Maura D, Galtier M, Le Bouguenec C, Debarbieux L. Virulent bacteriophages can target O104:H4 enteroaggregative *Escherichia coli* in the mouse intestine. Antimicrob Agents Chemother. 2012; 56(12): 6235–42. 10.1128/AAC.00602-12 23006754 PMC3497199

[13] Meader E, Mayer MJ, Steverding D, Carding SR, Narbad A. Evaluation of bacteriophage therapy to control *Clostridium difficile* and toxin production in an in vitro human colon model system. Anaerobe. 2013; 22: 25–30. 10.1016/j.anaerobe.2013.05.001 . 23685029

[14] Chhibber S, Gupta P, Kaur S. Bacteriophage as effective decolonising agent for elimination of MRSA from anterior nares of BALB/c mice. BMC Microbiol. 2014; 14: 212 10.1186/s12866-014-0212-8 . 25112504 PMC4236609

[15] Paisano AF, Spira B, Cai S, Bombana AC. *In vitro* antimicrobial effect of bacteriophages on human dentin infected with *Enterococcus faecalis* ATCC 29212. Oral Microbiol Immunol. 2004; 19(5): 327–30. 10.1111/j.1399-302x.2004.00166.x . 15327646

[16] Machuca P, Daille L, Vines E, Berrocal L, Bittner M. Isolation of a novel bacteriophage specific for the periodontal pathogen *Fusobacterium nucleatum* . Appl Environ Microbiol. 2010; 76(21): 7243–50. 10.1128/aem.01135-10 . 20851973 PMC2976222

[17] Phee A, Bondy-Denomy J, Kishen A, Basrani B, Azarpazhooh A, Maxwell K. Efficacy of bacteriophage treatment on *Pseudomonas aeruginosa* biofilms. J Endod. 2013; 39(3): 364–9. 10.1016/j.joen.2012.10.023 . 23402508

[18] Delisle AL, Guo M, Chalmers NI, Barcak GJ, Rousseau GM, Moineau S. Biology and genome sequence of *Streptococcus mutans* phage M102AD. Appl Environ Microbiol. 2012; 78(7): 2264–71. 10.1128/aem.07726-11 . 22287009 PMC3302630

[19] Van Der Ploeg JR. Genome sequence of *Streptococcus mutans* bacteriophage M102. FEMS microbiology letters. 2007; 275(1): 130–8. 10.1111/j.1574-6968.2007.00873.x 17711456

[20] Delisle AL, Rostkowski CA. Lytic bacteriophages of *Streptococcus mutans* . Curr Microbiol. 1993; 27(3): 163–7. 10.1007/bf01576015 . 23835749

[21] Shibata Y, Ozaki K, Seki M, Kawato T, Tanaka H, Nakano Y, et al Analysis of loci required for determination of serotype antigenicity in *Streptococcus mutans* and its clinical utilization. Journal of clinical microbiology. 2003; 41(9): 4107–12. 10.1128/jcm.41.9.4107-4112.2003 12958233 PMC193837

[22] Casey E, Mahony J, Neve H, Noben J-P, Bello FD, van Sinderen D. Genome and proteome analysis of bacteriophage Ldl1 reveals the existence of a novel phage group infecting *Lactobacillus delbrueckii* subsp. *lactis* . Appl Environ Microbiol. 2014; 81(4): 1319–26. 10.1128/aem.03413-14 PMC4309708 25501478

[23] Broussard Gregory W, Oldfield Lauren M, Villanueva Valerie M, Lunt Bryce L, Shine Emilee E, Hatfull Graham F. Integration-Dependent Bacteriophage Immunity Provides Insights into the Evolution of Genetic Switches. Mol Cell. 2013; 49(2): 237–48. 10.1016/j.molcel.2012.11.012 23246436 PMC3557535

[24] Baron F, Cochet M-F, Ablain W, Grosset N, Madec M-N, Gonnet F, et al Rapid and cost-effective method for micro-organism enumeration based on miniaturization of the conventional plate-counting technique. Lait. 2006; 86(3): 251–7.

[25] Boland AB, Buhr K, Giannouli P, van Ruth SM. Influence of gelatin, starch, pectin and artificial saliva on the release of 11 flavour compounds from model gel systems. Food Chem. 2004; 86(3): 401–11. 10.1016/j.foodchem.2003.09.015.

[26] Tunney MM, Ramage G, Field TR, Moriarty TF, Storey DG. Rapid colorimetric assay for antimicrobial susceptibility testing of *Pseudomonas aeruginosa* . Antimicrob Agents Chemother. 2004; 48(5): 1879–81. . 15105149 10.1128/AAC.48.5.1879-1881.2004 PMC400562

[27] Delcher AL, Harmon D, Kasif S, White O, Salzberg SL. Improved microbial gene identification with GLIMMER. Nucleic acids research. 1999; 27(23): 4636–41. . 10556321 10.1093/nar/27.23.4636 PMC148753

[28] Aziz RK, Bartels D, Best AA, DeJongh M, Disz T, Edwards RA, et al The RAST Server: rapid annotations using subsystems technology. BMC Genomics. 2008; 9: 75 10.1186/1471-2164-9-75 . 18261238 PMC2265698

[29] Altschul SF, Madden TL, Schaffer AA, Zhang J, Zhang Z, Miller W, et al Gapped BLAST and PSI-BLAST: a new generation of protein database search programs. Nucleic acids research. 1997; 25(17): 3389–402. . 9254694 10.1093/nar/25.17.3389 PMC146917

[30] Jones DT, Taylor WR, Thornton JM. The rapid generation of mutation data matrices from protein sequences. Comput Appl Biosci. 1992; 8(3): 275–82. . 1633570 10.1093/bioinformatics/8.3.275

[31] Tamura K, Stecher G, Peterson D, Filipski A, Kumar S. MEGA6: Molecular Evolutionary Genetics Analysis version 6.0. Mol Biol Evol. 2013; 30(12): 2725–9. 10.1093/molbev/mst197 . 24132122 PMC3840312

[32] Felsenstein J. Confidence limits on phylogenies: an approach using the bootstrap. Evolution. 1985; 39(4): 783–91. 10.2307/2408678 28561359

[33] Armau E, Bousque JL, Boue D, Tiraby G. Isolation of lytic bacteriophages for *Streptococcus mutans* and *Streptococcus sobrinus* . J Dent Res. 1998; 67: 121.

[34] Higuchi M, Rhee GH, Araya S, Higuchi M. Bacteriophage deoxyribonucleic acid-induced mutation of *Streptococcus mutans* . Infect Immun. 1977; 15(3): 938–44. . 870435 10.1128/iai.15.3.938-944.1977 PMC421463

[35] Bachrach G, Leizerovici-Zigmond M, Zlotkin A, Naor R, Steinberg D. Bacteriophage isolation from human saliva. Lett Appl Microbiol. 2003; 36(1): 50–3. . 12485342 10.1046/j.1472-765x.2003.01262.x

[36] Hitch G, Pratten J, Taylor PW. Isolation of bacteriophages from the oral cavity. Lett Appl Microbiol. 2004; 39(2): 215–9. 10.1111/j.1472-765X.2004.01565.x 15242464

[37] Nakano K, Ooshima T. Serotype classification of *Streptococcus mutans* and its detection outside the oral cavity. Future Microbiol. 2009; 4(7): 891–902. 10.2217/fmb.09.64 . 19722842

[38] Shibata Y, Yamashita Y, van der Ploeg JR. The serotype-specific glucose side chain of rhamnose-glucose polysaccharides is essential for adsorption of bacteriophage M102 to *Streptococcus mutans* . FEMS microbiology letters. 2009; 294(1): 68–73. 10.1111/j.1574-6968.2009.01546.x . 19493010

[39] van der Ploeg JR. Analysis of CRISPR in *Streptococcus mutans* suggests frequent occurrence of acquired immunity against infection by M102-like bacteriophages. Microbiol. 2009; 155(Pt 6): 1966–76. 10.1099/mic.0.027508-0 . 19383692

[40] Serbanescu MA, Cordova M, Krastel K, Flick R, Beloglazova N, Latos A, et al Role of the *Streptococcus mutans* CRISPR-Cas systems in immunity and cell physiology. J Bacteriol. 2015; 197(4): 749–61. 10.1128/jb.02333-14 . 25488301 PMC4334182

[41] Goldfarb T, Sberro H, Weinstock E, Cohen O, Doron S, Charpak-Amikam Y, et al BREX is a novel phage resistance system widespread in microbial genomes. EMBO J. 2015; 34(2): 169–83. 10.15252/embj.201489455 . 25452498 PMC4337064

[42] Mahony J, van Sinderen D. Current taxonomy of phages infecting lactic acid bacteria. Frontiers in microbiology. 2014; 5: 7 10.3389/fmicb.2014.00007 . 24478767 PMC3900856

[43] Wittebole X, De Roock S, Opal SM. A historical overview of bacteriophage therapy as an alternative to antibiotics for the treatment of bacterial pathogens. Virulence. 2014; 4(1): 1–10. 10.4161/viru.25991. PMC3916379 23973944

[44] O'Flaherty S, Ross RP, Meaney W, Fitzgerald GF, Elbreki MF, Coffey A. Potential of the polyvalent anti-*Staphylococcus* bacteriophage K for control of antibiotic-resistant staphylococci from hospitals. Appl Environ Microbiol. 2005; 71(4): 1836–42. 10.1128/AEM.71.4.1836-1842.2005 . 15812009 PMC1082512

[45] Ito T, Maeda T, Senpuku H. Roles of salivary components in *Streptococcus mutans* colonization in a new animal model using NOD/SCID.e2f1-/- mice. PLoS One. 2012; 7(2): e32063 10.1371/journal.pone.0032063 . 22363797 PMC3283720

[46] Decker E-M, Klein C, Schwindt D, von Ohle C. Metabolic activity of *Streptococcus mutans* biofilms and gene expression during exposure to xylitol and sucrose. In J Oral Sci. 2014; 6(4): 195–204. 10.1038/ijos.2014.38 PMC5153587 25059251

[47] Freires IA, Bueno-Silva B, Galvao LC, Duarte MC, Sartoratto A, Figueira GM, et al The Effect of essential oils and bioactive fractions on *Streptococcus mutans* and *Candida albicans* biofilms: A confocal analysis. Evid Based Complement Alternat Med. 2015; 2015: 871316 10.1155/2015/871316 . 25821503 PMC4363662

[48] O'Connor EB, O'Riordan B, Morgan SM, Whelton H, O'Mullane DM, Ross RP, et al A lacticin 3147 enriched food ingredient reduces *Streptococcus mutans* isolated from the human oral cavity in saliva. J Appl Microbiol. 2006; 100(6): 1251–60. 10.1111/j.1365-2672.2006.02856.x 16696672

[49] Wu CC, Lin CT, Wu CY, Peng WS, Lee MJ, Tsai YC. Inhibitory effect of *Lactobacillus salivarius* on *Streptococcus mutans* biofilm formation. Molecular oral microbiology. 2014; 30(1): 16–26. 10.1111/omi.12063 . 24961744

[50] Lin X, Chen X, Chen Y, Jiang W, Chen H. The effect of five probiotic lactobacilli strains on the growth and biofilm formation of *Streptococcus mutans* . Oral Dis. 2015; 21(1): e128–34. 10.1111/odi.12257 . 24806217

[51] Chan BK, Abedon ST, Loc-Carrillo C. Phage cocktails and the future of phage therapy. Future Microbiol. 2013; 8(6): 769–83. 10.2217/fmb.13.47 23701332

[52] Ly-Chatain MH. The factors affecting effectiveness of treatment in phages therapy. Frontiers in microbiology. 2014; 5: 51 10.3389/fmicb.2014.00051 . 24600439 PMC3927074

